# Association between illness perception and social alienation among maintenance hemodialysis patients: The mediating role of fear of progression

**DOI:** 10.1371/journal.pone.0301666

**Published:** 2024-04-02

**Authors:** Beisha Zhu, Hang Wu, Siyu Lv, Yulan Xu

**Affiliations:** 1 School of Nursing, Tongji Medical College, Huazhong University of Science and Technology, Wuhan, Hubei, P.R. China; 2 Union Hospital, Tongji Medical College, Huazhong University of Science and Technology, Wuhan, Hubei, P.R. China; 3 Tongji Hospital, Tongji Medical College, Huazhong University of Science and Technology, Wuhan, Hubei, P.R. China; Beijing University of Technology, CHINA

## Abstract

**Purpose:**

This study aimed to investigate the mediating role of fear of progression on illness perception and social alienation among maintenance hemodialysis (MHD) patients.

**Background:**

MHD is frequently accompanied by increased pain and complications such as itchy skin, chronic fatigue, and muscle spasms. Cardiovascular disease rates are also elevated among MHD patients, which can heighten their anxiety regarding prognosis and treatment discomfort. This chronic fear may severely impact social functioning, leading patients to withdraw from interpersonal interactions and experience heightened helplessness and loneliness. Further investigation is necessary to understand the factors behind the high level of social alienation in MHD patients and their underlying mechanisms.

**Design:**

A cross-sectional study guided by the STROBE.

**Methods:**

A convenience sample of 230 MHD patients were enrolled from January to May 2023. Data including demographic and clinical characteristics, illness perception, fear of progression, and social alienation were collected. Descriptive analysis and Pearson correlations were conducted using IBM SPSS version 25.0. The mediating effect was analyzed using Model 4 of the PROCESS macro for SPSS, with the Bootstrap method employed to assess its significance.

**Results:**

The score of social alienation in MHD patients was high, with illness perception and fear of progression both significantly correlated with social alienation. In the mediating effects model, illness perception can predict social alienation in MHD patients, and fear of progression use plays a part in mediating the process by which illness perception affects social alienation. The Kappa Squared (κ^2^) value of 21.9%, suggests a medium effect size.

**Conclusions:**

Illness perception directly predicts social alienation in MHD patients and exerts an indirect effect through the mediating role of fear of progression. Suggests that healthcare professionals should concentrate on MHD patients with high negative illness perceptions to alleviate their fear of progression, thereby decreasing the level of social alienation and enhancing their integration into society.

## Introduction

End-stage renal disease (ESRD), a progressive and debilitating condition characterized by the advanced deterioration of kidney function, presents a substantial and formidable health risk [1]. ESRD patients need to undergo kidney replacement therapy (KRT) for treatment, which is relied upon by 3.9 million people worldwide [2,3]. Among the various forms of KRT, maintenance hemodialysis (MHD) is the most widely used and accepted, accounting for about 69% of all KRT and 89% of all dialysis cases [4]. MHD has proven to be prolonging the lives of ESRD patients [5,6]. However, With the continuous advancement of the new model of modern biopsychosocial medicine [7], the prolongation of a patient’s life is no longer regarded as the only index for evaluating the treatment effect; and the nursing model should be transformed from the traditional therapeutic nursing to the nursing care model should also be changed from traditional curative care to comprehensive care, i.e., healthcare personnel should provide psychosocial services for patients while emphasizing on relieving their clinical symptoms, to better improve the quality of care [8]. That is to say, medical and nursing staff should not only emphasize relieving patients’ clinical symptoms but also provide patients with psychosocial services, to better improve their quality of life during the survival period.

According to the definition requirements of the World Health Organization (WHO), the quality of survival is a multidimensional concept, which includes four aspects: physiological, psychological, social functioning, and material status, while the diagnosis of ESRD and the treatment of MHD, in addition to causing physiological discomfort and pain to the patient, will also affect the patient’s psychological status and social functioning, leading to the patient’s physical and mental health, social participation, and other aspects of the situation is not optimistic [9]. MHD patients suffer from more complications than others, such as chronic pain [10,11], itchy skin [12], chronic fatigue [13] and muscle spasms [14], etc. Meanwhile, the incidence and mortality rates of cardiovascular diseases in MHD patients are higher than those in the general population [15], so the uncertainty of the disease prognosis, coupled with the pain caused by the treatment, can lead to feelings of worry and fear in patients. Chronic and excessive fear of disease progression affects patients’ social functioning [16], prompting MHD patients to choose to withdraw and distance themselves from daily interpersonal interactions, which in turn leads to helplessness and loneliness [17], that is, a state of “social alienation”.

Social alienation refers to the perception of negative treatment by the outside world when engaging in social interactions, leading to a sense of estrangement from others and society [18]. It encompasses subjective emotions such as loneliness and indifference, as well as objective behaviors such as rejection and avoidance. Studies have shown that social alienation was associated with an increase in poor health behaviors, including decreased physical activity, unhealthy dietary patterns, and increased suicidal ideation [19,20]. Unhealthy lifestyle choices and dietary patterns have been widely acknowledged as risk factors for a range of diseases, including cardiovascular disease [21–23]. Pertinently, these factors can also exert a significant impact on the management and control of kidney disease [24]. Research has shown that MHD patients suffer from extensive and significant social alienation [25], which has a severe impact on all aspects of their physical, mental, and social health. Therefore, there exists an immense significance in quantifying the level of social alienation among MHD patients and comprehensively scrutinizing its potential associations with various variables.

Illness perception (IP) is defined as individuals’ use of prior illness cognitions and experiences to analyze, regulate, and modify their current illness or threatened health state [26]. In the Common-Sense model of Self-Regulation (CSM) [27], both patients’ cognitive and affective perceptions of illness hold substantial sway over their overall well-being and emotional state, which subsequently exert influence on their adaptive coping mechanisms, including the inclination towards social withdrawal. The correlation between IP and social interaction has been demonstrated in many patients with chronic diseases. Prior investigations have established a compelling association between illness perception (IP) and social alienation across various patient cohorts, including those affected by interstitial lung disease [28], cancer [29,30], and nonepileptic seizures [31]. It is with this backdrop that we proffer a well-founded conjecture, hypothesizing an intrinsic correlation between IP and social alienation.

Fear of progression (FOP) is very common and has been the focus of research in recent years across a variety of chronic diseases [32,33]. In its essence, the term encompasses an individual’s instinctual and cognizant apprehension of the intricate ramifications of disease progression, spanning the realms of biology, psychology, and society [34]. Although moderate fear-induced defensive behaviors are beneficial to the patient’s physical health, prolonged and excessive fear can lead to adverse outcomes and induce social avoidance behaviors in patients [35]. Studies have demonstrated that heightened concerns regarding disease progression predict higher levels of psychosocial distress in patients [36]. However, as far as our knowledge extends, the possible mediating role of FOP between IP on social alienation has not been explored in the current research. Therefore, we hypothesized that FOP mediates IP and social alienation.

**Fig 1:**
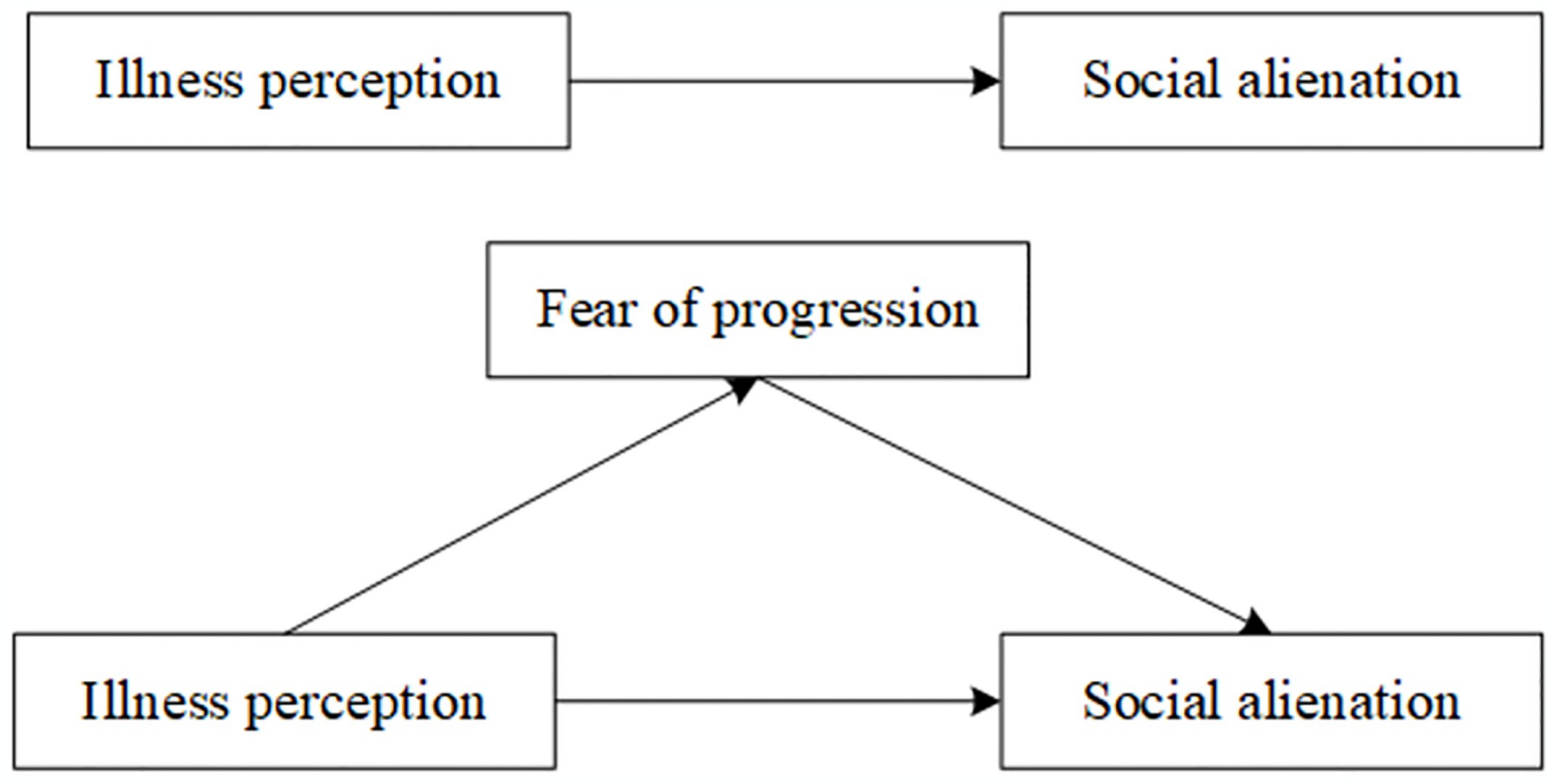
Hypothesized model.

Taking into account the above overview, our study set out to investigate Chinese MHD patients, quantifying the extent of social alienation in MHD patients and identifying potential mechanisms involved. First, we hypothesized that MHD patients have high levels of social alienation. Second, we hypothesized a correlation between IP and social alienation. Finally, we hypothesized that FOP mediates IP and social alienation. Therefore, we propose the following research hypotheses, as illustrated in Fig 1.

## Methods

### Design

The study employed a cross-sectional correlational study survey design and complied with the STROBE (Strengthening the Reporting of Observational Studies in Epidemiology) checklist.

### Sample

We used a convenience sampling method to select MHD patients who attended a hemodialysis center in three tertiary hospitals in Wuhan, Hubei province, from January 2023 to May 2023.

The criteria for participant inclusion in this study encompassed the following key aspects: (1) Patients aged 18 years or above; (2) Patients who received regular hemodialysis for at least 3 months; (3) Patients possessing written and oral reading and communication abilities; (4) Patients who provided informed consent and voluntarily participated in this study. The participant exclusion criteria encompassed the following aspects: (1) Patients presenting with other significant and life-threatening illnesses; (2) Patients diagnosed with severe cognitive impairment or mental disorders; (3) Patients who dropped out or were unable to answer a complete questionnaire.

### Sample size

The sample size was calculated using G-Power 3.1.9.7. The calculations were based on an α level of 0.05, power of 0.95, medium effect size of 0.25, and considering a 20% sample loss rate. The minimum required sample size was determined to be 168 cases. A total of 250 questionnaires were distributed in this study and 230 questionnaires were validly returned with a validity rate of 92%, which satisfied the minimum sample size required for this study.

### Variables and instruments

#### Demographic and clinical characteristics

Demographic data includes MHD patients’ age, gender, education level, marital status, and monthly income. Clinical information included primary cause, number of comorbidities, duration of hemodialysis, and frequency of dialysis frequency.

#### Illness perception

The assessment of IP was performed using the Brief Illness Perception Questionnaire (BIPQ) [37]. Developed by Broadbent [38], the BIPQ was employed to evaluate patients’ cognitive and emotional reactions toward their illness. The questionnaire comprised nine items, each rated on a scale of 0 to 10, except for the item about the perceived cause of the disease. The total score ranged from 0 to 80, with higher scores signifying heightened negative perceptions of the disease and greater severity of its symptoms. Item 9 of the questionnaire entailed an open-ended inquiry, wherein patients were prompted to pinpoint the three most substantial factors contributing to their illness. Widely used in the MHD patients, this scale has demonstrated a Cronbach’s alpha coefficient of 0.757 [39]. In the present study, the scale demonstrated a Cronbach’s α coefficient of 0.829, indicating good internal consistency.

#### Fear of progression

The assessment of FOP was performed using the Fear of Progression Questionnaire-Short Form (FoP-Q-SF) [40]. Developed by Mehner, the FoP-Q-SF was specifically designed to evaluate the degree of fear experienced by patients with chronic diseases regarding disease progression or relapse. The scale employed in this study comprises 12 items and encompasses two dimensions: physical health and socio-family aspects. Each item was evaluated using a five-point Likert scale, spanning from “never” to “always,” generating a composite score ranging from 12 to 60. Higher scores are indicative of an intensified apprehension regarding the progression of the disease. Notably, if the total score equals or exceeds 34, it signifies the presence of psychological dysfunction in the patient. This scale is currently widely utilized among renal dialysis patients in China, demonstrating a Cronbach’s alpha coefficient of 0.895 [41]. In the present study, the scale demonstrated a Cronbach’s α coefficient of 0.727, indicating good internal consistency.

#### Social alienation

The assessment of Social alienation was performed using the General Social Alienation Scale (GSAS) [42]. The GSAS, developed by Jessor [42], was utilized to assess the degree of social alienation by individuals. The scale comprises a total of 15 items, encompassing four distinct dimensions: social alienation, powerlessness, self-alienation, and meaninglessness. Each item was assessed using a 4-point Likert scale, spanning from “strongly disagree” to “strongly agree,” with a range of 1 to 4. As a result, the overall score ranged from 15 to 60, with higher scores denoting heightened levels of alienation. The GSAS is widely employed among MHD patients in China, demonstrating a Cronbach’s alpha coefficient of 0.805 [43]. In the present study, the scale demonstrated a Cronbach’s α coefficient of 0.831, indicating good internal consistency.

### Data collection

Structured paper-and-pencil questionnaires were utilized for data collection, encompassing four distinct sections: demographic and clinical information, IP, FOP, and social alienation. We tried to distribute the questionnaires at times when they were not fatigued, such as before the start of hemodialysis or within 1 to 2 hours after the start. For patients who were not convenient to fill in the questionnaire, the investigator asked for and recorded each item. We distributed, monitored, and collected the questionnaires on-site, actively addressing inquiries raised by the study participants. Subsequently, we inspected the retrieved questionnaires to ensure their completeness, considering any missing responses as invalid for patients who abandoned questionnaire completion midway through the process. The data underlying this research can be found in the S1 Data.

### Data analysis

Statistical analyses were performed employing IBM SPSS version 25.0. All statistical tests are two-way (α = 0.05). Count data were described by frequency and composition ratio, whereas measurement data that conform to the normal distribution were described by the mean and standard deviation (SD) and compared between groups using independent samples t-test and one-way ANOVA. The measurement data that did not conform to a normal distribution were described by the median and quartiles, and the Mann–Whitney U test and Kruskal–Wallis H test was used for comparison between groups. To investigate the relationships between IP, FOP, and social alienation, the Pearson correlation coefficient was utilized.

Mediation analysis was performed using Model 4 of the PROCESS macro for SPSS [44]. In our statistical analyses, social alienation was used as the dependent variable, IP as the independent variable, and FOP as the mediating variable. We controlled for the following demographic variables and clinical information: age, gender (0 = male, 1 = female), education level (four dummy-coded variables), marital status (two dummy-coded variables), monthly income (three dummy-coded variables), primary cause (four dummy-coded variables), number of comorbidities (three dummy-coded variables), duration of hemodialysis (three dummy-coded variables), and dialysis frequency (two dummy-coded variables).

The mediating role of FOP was examined using the bootstrap resampling approach with 5000 bootstrap samples and a 95% confidence interval (CI) around the standardized estimate. The point estimates and 95% CI of the direct effects, indirect effects, and total effects were assessed using bootstrapping with 5,000 simulations. A statistically significant effect was determined if the 95% CI interval excluded zero. Effect sizes for mediated effects were calculated using Kappa Squared (κ^2^), as recommended by Preacher and Kelley [45], with values of 0.01 considered small, 0.09 considered medium, and 0.25 considered large effects.

### Ethics statement

The study received ethical approval from the Medical Ethics Committee of Tongji Medical College of Huazhong University of Science and Technology, China, under the reference [2022] Ethics (S230). The researchers provided a detailed explanation of the study’s objectives to the participants, ensuring their understanding of the research purpose. Additionally, a commitment was made to anonymize all data collected. Before participating in the study, each participant gave written informed consent. Significantly, participants were provided with unequivocal assurance regarding their absolute autonomy to discontinue their involvement in the study at any juncture.

## Results

### Characteristics of the participants

A total of 230 patients with MHD participated in this study. The ages of the participants ranged from 24 to 88 years; the median age was 57 (44, 74) years. The male gender constituted the majority of the participants (60.0%), while individuals with a high school education completion accounted for 30.0% of the sample. Furthermore, a significant proportion of the participants were married (75.7%), and their monthly income fell within the range of 2001–4000 RMB (30.9%). Additionally, 34.8% of the participants were suffering from two kinds of chronic comorbidities. Of these participants, 28.7% had kidney disease due to hypertension. Regarding the duration of hemodialysis, a total of 64 participants (27.8%) received hemodialysis treatments ranging from 1 to 3 years. The frequency of dialysis among the participants was mainly three times a week.

### The relationships among IP, FOP, and social alienation

Table 1 showed that IP and FOP were both positively correlated with social alienation. Additionally, a statistically significant positive correlation was ascertained between IP and FOP.

**Table 1:** The relationships among IP, FOP, and social alienation.

Variables	Mean (SD)	Illness perception	Fear of progression	Social alienation
Illness perception	45.50±6.40	1		
Fear of progression	35.99±5.93	0.475*	1	
Social alienation	39.90±6.22	0.652*	0.617*	1

*p < 0.001.

### The mediating role of FOP in the relationship between IP and social alienation

The results of the mediating role are presented in Tables 2 and 3. Within the framework of our study’s mediation model, social alienation was considered the dependent variable, IP was the independent variable, and FOP was the mediating variable. To mitigate potential confounding effects and enhance the statistical power of the analysis, several covariates, namely age, gender, education level, marital status, monthly income, Primary cause, number of comorbidities, duration of hemodialysis, and dialysis frequency, were included in the ‘Covariates module during the bootstrap procedure. This ensured that these variables were controlled to minimize their impact on the results.

**Table 2:** Mediation model tests with control variables.

Investigation content	Outcome variables								
	Social alienation			Social alienation			Fear of progression		
	*β*	*t*	*p*	*β*	*t*	*p*	*β*	*t*	*p*
Predictor variable									
Age	0.003	0.982	0.328	0.004	1.136	0.257	0.003	0.581	0.562
Gender	-0.016	-0.178	0.859	-0.024	-0.239	0.811	-0.023	-0.186	0.853
Education level									
Junior high school^(^^1^^)^	0.071	0.385	0.700	0.098	0.482	0.630	0.080	0.317	0.751
High school^(^^1^^)^	-0.025	-0.138	0.891	-0.046	-0.228	0.820	-0.062	-0.248	0.805
College for professional training^(^^1^^)^	-0.180	-0.877	0.381	-0.258	-1.154	0.250	-0.238	-0.856	0.393
Bachelor’s degree and above^(^^1^^)^	-0.458	-2.124	0.035	-0.489	-2.070	0.040	-0.091	-0.311	0.756
Marital status									
Married^(^^2^^)^	-0.162	-1.049	0.295	0.274	-1.630	0.145	0.337	-1.623	0.106
Divorced/widowed^(^^2^^)^	-0.033	-0.177	0.860	0.039	-0.188	0.851	-0.016	-0.064	0.949
Monthly income									
2001–4000 ^(^^3^^)^	-0.196	-1.568	0.119	-0.238	-1.739	0.084	-0.123	-0.739	0.461
4001–6000 ^(^^3^^)^	0.008	0.059	0.953	-0.340	-0.274	0.785	-0.145	-0.800	0.425
≥6001 ^(^^3^^)^	-0.227	-1.391	0.166	-0.271	-1.522	0.130	-0.134	-0.606	0.545
Primary cause									
Diabetes^(^^4^^)^	-0.161	-1.110	0.268	-0.112	-0.706	0.481	0.150	0.76	0.448
Hypertension^(^^4^^)^	0.013	0.103	0.918	0.074	0.557	0.578	0.186	1.130	0.260
Polycystic kidney^(^^4^^)^	-0.312	-1.746	0.082	-0.236	-1.213	0.227	0.229	0.947	0.345
Others^(^^4^^)^	-0.289	-2.210	0.028	-0.301	-2.104	0.037	-0.036	-0.201	0.841
Number of comorbidities									
1^(^^5^^)^	-0.015	-0.096	0.923	0.058	0.337	0.736	0.224	1.039	0.300
2^(^^5^^)^	0.087	0.552	0.582	0.092	0.534	0.594	0.015	0.072	0.943
≥3^(^^5^^)^	0.173	0.983	0.327	0.223	1.164	0.246	0.153	0.643	0.521
Duration of hemodialysis									
1-3^(^^6^^)^	-0.012	-0.080	0.936	-0.136	-0.868	0.387	-0.377	-1.938	0.054
3-5^(^^6^^)^	-0.238	-1.457	0.147	-0.432	-2.464	0.015	-0.590	-2.707	0.007
5-10^(^^6^^)^	-0.330	-2.260	0.025	-0.469	-2.967	0.003	-0.421	-2.142	0.033
≥10^(^^6^^)^	-0.189	-1.139	0.256	-0.303	-1.676	0.095	-0.344	-1.533	0.127
Dialysis frequency									
2.5^(^^7^^)^	0.195	1.502	0.135	0.313	2.227	0.027	0.358	2.049	0.042
≥3^(^^7^^)^	0.221	1.841	0.067	0.391	3.050	0.003	0.515	3.234	0.001
Illness perception	0.372	6.598	<0.001	0.496	8.560	<0.001	0.375	5.217	<0.001
Fear of progression	0.330	6.393	<0.001						
Fit indices									
F	14.215			10.999			4.252		
R	0.803			0.758			0.585		
R^2^	0.646			0.574			0.343		
P	<0.001			<0.001			<0.001		

All path coefficient was standardized.

^(1)^ Primary school or below as reference;

^(2)^ Single as reference;

^(3)^ ≤2000 as reference;

^(4)^ Chronic nephritis as reference;

^(5)^ 0 as reference;

^(6)^ <1 as reference;

^(7)^ 2 as reference.

**Table 3:** Mediating, direct, indirect, and total effects of IP, FOP, and social alienation.

Variables	β	SE	95% CI	
			Lower	Upper
Total effect	0.496	0.058	0.381	0.610
Direct effect	0.372	0.056	0.261	0.483
Indirect effect	0.124	0.035	0.066	0.203

All path coefficient was standardized.

**Fig 2:**
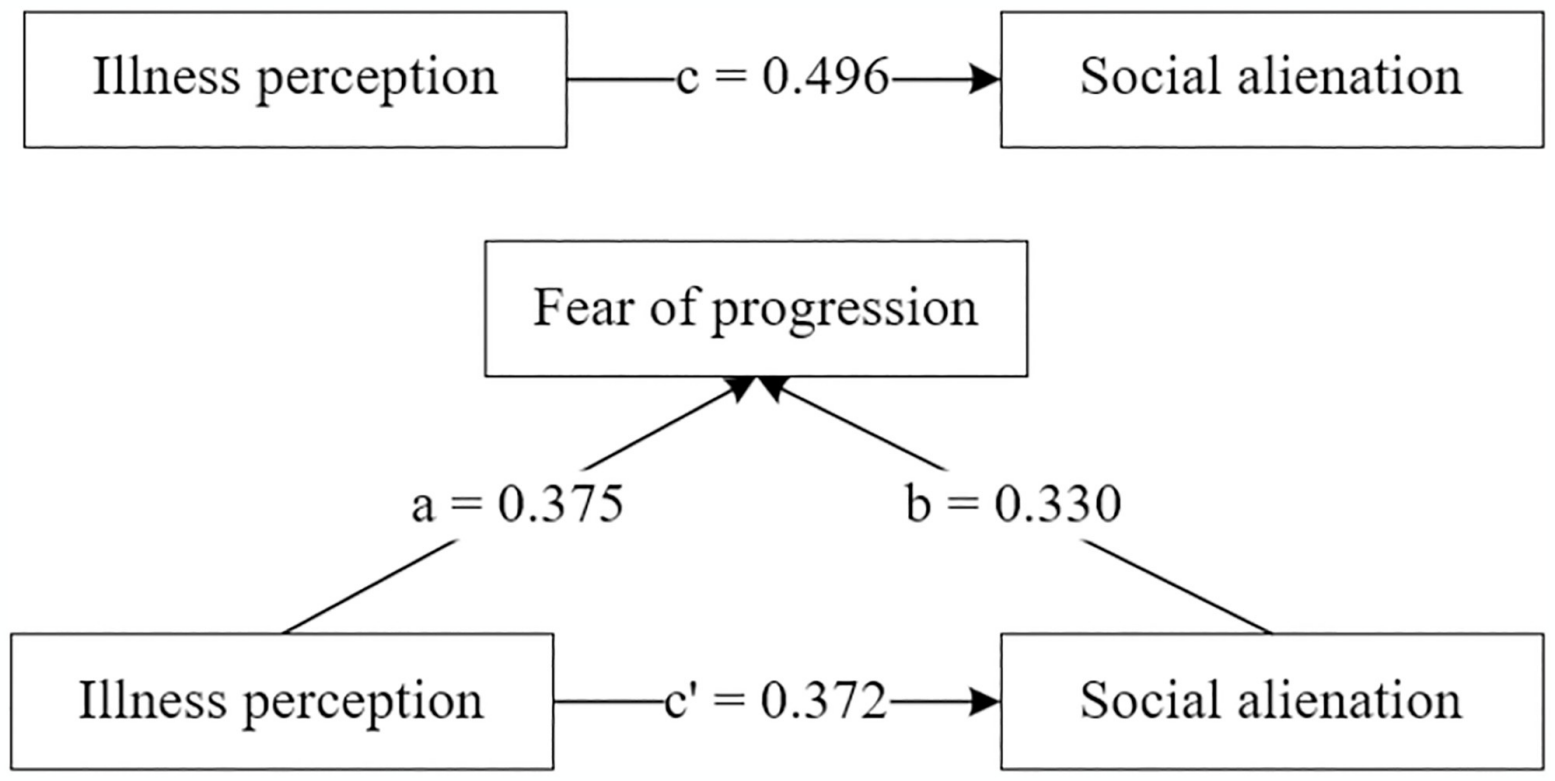
The mediating role of FOP on the relationship between IP and social alienation.

The results indicate that there was a significant total effect (path c) of IP on social alienation, indicating a positive relationship between IP and social alienation. Moreover, significant coefficients were found for paths a and b, indicating a positive relationship between IP and FOP, as well as a positive correlation between FOP and social alienation. Additionally, the point estimate of the indirect effects (a * b) connecting IP to social alienation through FOP according to our analysis. We documented Kappa Squared values of 21.9% (κ2 = 0.219), suggesting the medium effect of the mediation by FOP in the relationship between IP and social alienation. A graphical representation of the final mediation model is displayed in Fig 2.

### Comparing social alienation scores of participants with different characteristics

As illustrated in Table 4, notable variations in the total score of social alienation were observed across different factors, including education level, marital status, monthly income, number of comorbidities, duration of hemodialysis, and dialysis frequency. However, there was no significance among different genders, and primary causes (*p* > 0.05).

**Table 4:** The difference between different groups of social alienation (*N* = 230).

Variables	*N* (%)	Social alienation score	*t/F/Z*	*p*
Gender			-1.963 ^(^^1^^)^	0.051
Male	138(60.0)	39.25±6.39		
Female	92 (40.0)	40.88±5.85		
Educational level			10.283^(^^2^^)^	<0.001
Primary school or below	18 (7.8)	44.11±3.72		
Junior high school	58 (25.2)	42.12±5.93		
High school	69 (30.0)	40.43±6.00		
College for professional training	45 (19.6)	37.84±5.57		
Bachelor’s degree and above	40 (17.4)	36.18±6.07		
Marital status			5.162 ^(^^2^^)^	0.006
Single	27 (11.7)	41.52±5.59		
Married	174 (75.7)	39.18±6.29		
Divorced/widowed	29 (12.6)	42.69±5.39		
Monthly income (RMB, yuan)			9.206 ^(^^2^^)^	<0.001
≤2000	50 (21.7)	41.38±5.82		
2001–4000	71 (30.9)	41.04±5.65		
4001–6000	62 (27.0)	40.42±6.34		
≥6001	47 (20.4)	35.91±5.82		
Primary cause			1.523^(^^2^^)^	0.196
Chronic nephritis	58 (25.2)	40.98±5.83		
Diabetes	33 (14.3)	40.12±7.55		
Hypertension	66 (28.7)	40.23±6.64		
Polycystic kidney	19 (8.3)	37.37±5.35		
Others	54 (23.5)	39.09±5.48		
Number of comorbidities			12.214^(^^2^^)^	<0.001
0	25 (10.9)	36.76±6.21		
1	71 (30.9)	38.34±5.51		
2	80 (34.8)	39.65±5.45		
≥3	54 (23.5)	43.78±6.49		
Duration of hemodialysis (years)			11.403^(^^3^^)^	0.022
<1	36 (15.7)	43.50 (36.25, 47.00)		
1–3	64 (27.8)	41.00 (35.00, 46.00)		
3–5	38 (16.5)	39.50 (36.75, 42.25)		
5–10	59 (25.7)	39.00 (34.00, 42.00)		
≥10	33 (14.3)	38.00 (33.00, 42.50)		
Dialysis frequency (times/week)			11.440^(^^2^^)^	<0.001
2	53 (23.0)	36.51±6.36		
2.5	64 (27.8)	40.47±5.28		
≥3	113 (49.1)	41.17±6.12		

^(1)^ t-value;

^(2)^ H-value;

^(3)^ Z-value.

## Discussion

Social alienation presents a common concern in the realm of mental health among MHD patients. However, the examination of FOP as a mediating factor in the relationship between IP and social alienation in MHD patients has remained unexplored in prior investigations. As evident from our current study, the results substantiate our initial hypothesis that there exists a positive correlation between IP, FOP, and social alienation among MHD patients. Furthermore, our findings suggest that FOP plays a mediating function between IP and feelings of social alienation. In essence, MHD patients with negative disease perceptions are inclined to harbor apprehensions regarding the progression of their condition, which, in turn, contributes to amplified levels of social alienation.

### IP is positively correlated with social alienation

Similar to previous research [46,47], this study revealed a positive correlation between IP and social alienation. Illness cognition is a key factor in the interaction between psychology and illness. It can affect the patient’s psychological state and determine his or her level of self-regulation [48]. Positive disease awareness is conducive to enhancing their awareness of preventive health care and promoting medical compliance behaviors, thereby improving patients’ health and long-term quality of life [49–52]. However, the stronger the individual’s negative disease perception, the more severe the symptoms they experience, which may aggravate the patient’s negative emotions, reduce the confidence in coping with stressful events [19], reduce the individual’s willingness to contact the surrounding things and the environment, and increase social alienation feel. CSM shows that as the disease and treatment progress, an individual’s disease perception manifests itself as a dynamic process of constant modification and adjustment [53].

### FOP is positively correlated with social alienation

Consistent with previous research [54], this study revealed a positive correlation between FOP and social alienation. The theoretical model for fear of cancer recurrence (FCR) suggests that the patient’s experience of the illness generates FOP, which, in turn, leads to other negative emotions [55]. A moderate level of fear can act as a motivating factor, encouraging patients to adopt healthy behaviors and positive treatment approaches [56]. However, excessive fear functions as a strong inhibitor, putting patients in a negative state [57] when confronted with their illness and decreasing their quality of life [58,59]. Researchers have found that individuals with lower FOP are more able to consciously control their social life activities and consider all sides of the disease, treatment, life, and work, resulting in less social alienation [33]. Long-term excessive fear can cause chronic suffering [60], affecting patients’ quality of life and social functioning. FOP is one of the most prominent psychological distresses in MHD patients [61], they may fear that the disease may be worse, require more complex treatment, or be at risk for complications, and this uncertainty may have a significant negative impact on their lives, leading to worry and anxiety about participating in social activities and increased social alienation [62]. These results suggest that actively reducing FOP is an important way to reduce social alienation.

### FOP as a mediator between IP and social alienation

The purpose of this study was to investigate the effects of IP on social alienation and examine the underlying mediating mechanism involving FOP. Noteworthy findings emerged, affirming our original hypothesis as FOP played a significant mediator in the association between IP and social alienation.

Our findings suggest that patients who report negative IP have higher levels of FOP, which in turn leads to higher levels of social alienation. Especially in the early stages of hemodialysis treatment, patients have insufficient knowledge of the disease and hemodialysis and are fearful of complications [34,63,64], which causes them to overly focus on their diseases, resulting in a significant reduction in social activities and increased social alienation. Previous research has confirmed that negative disease perception is an internal stimulus and a significant predictor of higher fear of disease progression [65]. More specifically, MHD patients with a positive and correct conception of the disease can better regulate their fears during the course of the disease progression [66]. In other words, when people with MHD develop negative and discouraging perceptions of the disease, they may fear the severity and progression of the disease and doubt their health, which can lead to an increase in mental stress, negative emotions, and anxiety, fear, and depression [67]. These psychological reactions and emotional experiences may influence patients’ well-being and their social behaviors [68], causing them to become more withdrawn and avoid social situations, leading to an increased level of social alienation.

### The level of social alienation among MHD patients

In this study, it was found that the level of social alienation in MHD patients was identified to be moderately elevated, with a mean total score of 39.90 ± 6.22, which is similar to the results of previous research [25]. There are several reasons why MHD patients have moderately high levels of social alienation. First, due to the need for regular dialysis treatments in hospitals two to three times a week and frequent travel between hospitals and their homes, MHD patients experience a change in their social roles, inability to work and socialize normally, and limited participation in social activities, which can lead to social alienation [69,70]. Second, as a result of long-term hemodialysis, MHD patients’ physical function, muscle strength, and functional motor ability have declined, their social function has gradually weakened, and they have become more dependent on their caregivers for support and assistance, which has brought double pressure on the patients’ body and psychology, and exacerbated social alienation. Lastly, MHD patients have high rates of cardiovascular events and mortality [15,71], and patients have negative perceptions of the disease [72], leading to fear of disease progression and increased social alienation.

### Practical implications

The findings of this study have important practical implications. On the one hand, the study’s exploration of how IP influences social alienation through FOP offers insights into the psychological experiences of MHD patients. This understanding is crucial for fostering psychological resilience in patients, enabling them to better navigate the challenges of their illness. When patients adopt a more positive perspective on their condition and experience reduced social alienation, this can lead to increased treatment adherence, potentially improving overall health outcomes [43,73]. Moreover, numerous studies have shown a strong correlation between social functioning and positive emotional state [74–76]. By reducing social alienation among MHD patients, it is possible to enhance their experience of positive emotions and well-being, such as happiness, contentment, and gratitude, which are integral to contributing to positive psychology and to improving mental health and overall quality of life [77,78].

On the other hand, the study’s results contribute to revealing the underlying mechanisms of the psychological state of MHD patients, Providing a theoretical foundation for personalized interventions for healthcare professionals to improve patients’ sense of social alienation. For instance, interventions such as cognitive restructuring to modify illness perceptions [79] and reduce the impact of FOP [80], along with specialized health education materials that deepen patients’ understanding of their condition and treatment, can be effective [78,81]. Additionally, specialized psychosocial interventions [82,83], including psychotherapeutic Support, relaxation-based interventions, and group therapy sessions, can assist patients in reshaping their thought processes, alleviating anxiety about disease progression, and subsequently reducing the sense of social alienation.

Furthermore, the use of digital tools and telemedicine platforms allows for the establishment of online support systems, providing patients with real-time assistance. These systems can include databases of medical information, online consultation forums, and virtual support groups, helping to bridge geographical distances and reduce social disconnection [84–86]. Moreover, implementing continuous monitoring of patients’ physical and mental health, with regular reassessments of their illness perceptions and psychosocial needs, can ensure that care plans are adapted as necessary [9,87]. For patients experiencing severe psychological distress or significant functional limitations, accessible, intensive, and long-term professional support should be integrated into their care plans [88].

### Limitations

This study has certain theoretical and practical significance, but it also has some limitations. Firstly, this study was a cross-sectional design, thus, it cannot infer a causal relationship between IP, FOP, and social alienation. Secondly, this study employed convenience sampling and self-reporting methods for data collection, which may introduce potential biases such as selection bias and reporting bias. Therefore, it is important to exercise caution in interpreting the results of this study. In future research, it is recommended to employ a combination of self-evaluation and external evaluations for data collection and consider using different sampling methods to validate the findings. Additionally, given the multifaceted nature of factors influencing the explanatory variables, it is important to acknowledge that IP, while analyzed in this study, represents only one aspect influencing patients’ social alienation. Consequently, the developed mediation model should not be considered the sole mediation model, and it is essential to explore and test various other explanatory and mediating variables in future investigations.

## Conclusion

To sum up, this study showed that MHD patients had moderately high levels of social alienation, that IP was a positive predictor of social alienation, that FOP was a positive predictor of social alienation, and that FOP played a mediating role between IP and social alienation in MHD patients. The findings suggest that reducing negative perceptions of the disease can reduce patients’ fear of disease progression, thereby improving their social functioning and quality of life. Therefore, we suggest that disease cognition and fear assessment should be a routine task for MHD specialist nurses to help patients with more negative disease cognition in early stage to adjust themselves in time, to face the disease and treatment correctly, to reduce the fear of disease and complications, to promote participation in social activities, and to improve the quality of life of MHD patients.

## Supporting information

S1 Data (XLSX)

## References

[1] Gupta R, Woo K, Yi JA. Epidemiology of end-stage kidney disease. Semin Vasc Surg. 2021;34(1):71–8. doi: 10.1053/j.semvascsurg.2021.02.010 33757639 PMC8177747

[2] Jager KJ, Kovesdy C, Langham R, Rosenberg M, Jha V, Zoccali C. A single number for advocacy and communication-worldwide more than 850 million individuals have kidney diseases. Kidney Int. 2019;96(5):1048–50. doi: 10.1016/j.kint.2019.07.012 31582227

[3] Kramer A, Pippias M, Noordzij M, Stel VS, Andrusev AM, Aparicio-Madre MI, et al. The European Renal Association-European Dialysis and Transplant Association (ERA-EDTA) Registry Annual Report 2016: a summary. Clin Kidney J. 2019;12(5):702–20. doi: 10.1093/ckj/sfz011 31583095 PMC6768305

[4] Bello AK, Okpechi IG, Osman MA, Cho Y, Htay H, Jha V, et al. Epidemiology of haemodialysis outcomes. Nat Rev Nephrol. 2022;18(6):378–95. doi: 10.1038/s41581-022-00542-7 35194215 PMC8862002

[5] Wang F, Yang C, Long J, Zhao X, Tang W, Zhang D, et al. Executive summary for the 2015 Annual Data Report of the China Kidney Disease Network (CK-NET). Kidney Int. 2019;95(3):501–5. doi: 10.1016/j.kint.2018.11.011 30784660

[6] Betiru EA, Mamo E, Jara Boneya D, Adem A, Abebaw D. Survival Analysis and Its Predictors Among Hemodialysis Patients at Saint Paul Hospital Millennium Medical College and Myungsung Christian Medical Center in Addis Ababa, Ethiopia, 2021. Int J Nephrol Renov. 2023;16:59–71. doi: 10.2147/IJNRD.S401022 36875008 PMC9983441

[7] Engel GL. The need for a new medical model: a challenge for biomedicine. Science. 1977;196(4286):129–36. doi: 10.1126/science.847460 847460

[8] Wade DT, Halligan PW. The biopsychosocial model of illness: a model whose time has come. Clin Rehabil. 2017;31(8):995–1004. doi: 10.1177/0269215517709890 28730890

[9] Finnegan-John J, Thomas VJ. The psychosocial experience of patients with end-stage renal disease and its impact on quality of life: findings from a needs assessment to shape a service. Isrn Nephrology. 2013;2013:308986. doi: 10.5402/2013/308986 24959536 PMC4045426

[10] Marzouq MK, Samoudi AF, Samara A, Zyoud SH, Al-Jabi SW. Exploring factors associated with pain in hemodialysis patients: a multicenter cross-sectional study from Palestine. Bmc Nephrol. 2021;22(1):96. doi: 10.1186/s12882-021-02305-1 33731036 PMC7972237

[11] Rao Q, Zeng J, Wang S, Hao J, Jiang M. Chronic Pain and Quality of Life in Maintenance Hemodialysis Patients in China: A Multicenter, Cross-Sectional Study. J Pain Res. 2022;15:147–57. doi: 10.2147/JPR.S345610 35082527 PMC8784256

[12] Li W, Yin Y, Chen H, Wang X, Yun H, Li H, et al. Curative effect of neutral macroporous resin hemoperfusion on treating hemodialysis patients with refractory uremic pruritus. Medicine. 2017;96(12):e6160. doi: 10.1097/MD.0000000000006160 28328802 PMC5371439

[13] Bossola M, Hedayati SS, Brys ADH, Gregg LP. Fatigue in Patients Receiving Maintenance Hemodialysis: A Review. American Journal of Kidney Diseases: The Official Journal of the National Kidney Foundation. 2023;82(4):464–80. doi: 10.1053/j.ajkd.2023.02.008 37187283 PMC11571972

[14] Raja SM, Seyoum Y. Intradialytic complications among patients on twice-weekly maintenance hemodialysis: an experience from a hemodialysis center in Eritrea. Bmc Nephrol. 2020;21(1):163. doi: 10.1186/s12882-020-01806-9 32370756 PMC7201639

[15] Yang M, Yang Y, Xu Y, Wu Y, Lin J, Mai J, et al. Development and Validation of Prediction Models for All-Cause Mortality and Cardiovascular Mortality in Patients on Hemodialysis: A Retrospective Cohort Study in China. Clin Interv Aging. 2023;18:1175–90. doi: 10.2147/CIA.S416421 37534232 PMC10392814

[16] Wang Y, Yu Q, Zeng Z, Yuan R, Wang R, Chen J, et al. Predictors of fear of diabetes progression: A multi-center cross-sectional study for patients self-management and healthcare professions education. Front Public Health. 2022;10:910145. doi: 10.3389/fpubh.2022.910145 36600932 PMC9806215

[17] Nakajima C, Tomida K, Shimoda T, Kawakami A, Shimada H. Association between willingness to participate in physical and social activities and loneliness in older adults: A stratified analysis by social isolation status. Arch Gerontol Geriat. 2023;116:105216. doi: 10.1016/j.archger.2023.105216 37782967

[18] Matusitz J. Social Alienation.: Springer International Publishing; 2020. p. 103–33.

[19] Holt-Lunstad J, Smith TB, Baker M, Harris T, Stephenson D. Loneliness and Social Isolation as Risk Factors for Mortality. Perspect Psychol Sci. 2015;10(2):227–37. doi: 10.1177/1745691614568352 25910392

[20] Kanbay M, Tanriover C, Copur S, Peltek IB, Mutlu A, Mallamaci F, et al. Social isolation and loneliness: Undervalued risk factors for disease states and mortality. Eur J Clin Invest. 2023;53(10):e14032. doi: 10.1111/eci.14032 37218451

[21] Chareonrungrueangchai K, Wongkawinwoot K, Anothaisintawee T, Reutrakul S. Dietary Factors and Risks of Cardiovascular Diseases: An Umbrella Review. Nutrients. 2020;12(4). doi: 10.3390/nu12041088 32326404 PMC7231110

[22] Shi Z, Ganji V. Dietary patterns and cardiovascular disease risk among Chinese adults: a prospective cohort study. Eur J Clin Nutr. 2020;74(12):1725–35. doi: 10.1038/s41430-020-0668-6 32506113

[23] Zampelas A, Magriplis E. Dietary patterns and risk of cardiovascular diseases: a review of the evidence. The Proceedings of the Nutrition Society. 2020;79(1):68–75. doi: 10.1017/S0029665119000946 31250769

[24] Danaei G, Ding EL, Mozaffarian D, Taylor B, Rehm J, Murray CJL, et al. The preventable causes of death in the United States: comparative risk assessment of dietary, lifestyle, and metabolic risk factors. Plos Med. 2009;6(4):e1000058. doi: 10.1371/journal.pmed.1000058 19399161 PMC2667673

[25] Qin J, Zong X, Gao H. Investigation on the status quo of social alienation in maintenance hemodialysis patients and its correlation with self disclosure and social support. Chin J Prac Nurs. 2023(39):119–25.

[26] Broadbent E, Wilkes C, Koschwanez H, Weinman J, Norton S, Petrie KJ. A systematic review and meta-analysis of the Brief Illness Perception Questionnaire. Psychol Health. 2015;30(11):1361–85. doi: 10.1080/08870446.2015.1070851 26181764

[27] Leventhal H, Phillips LA, Burns E. The Common-Sense Model of Self-Regulation (CSM): a dynamic framework for understanding illness self-management. J Behav Med. 2016;39(6):935–46. doi: 10.1007/s10865-016-9782-2 27515801

[28] Liu Q, Qin T, Hu B, Zhao Y, Zhu X. Relationship between illness perception, fear of progression and quality of life in interstitial lung disease patients: A cross-sectional study. J Clin Nurs. 2021;30(23–24):3493–505. doi: 10.1111/jocn.15852 33998090

[29] Aydın Sayılan A, Demir Doğan M. Illness perception, perceived social support and quality of life in patients with diagnosis of cancer. Eur J Cancer Care. 2020;29(4):e13252. doi: 10.1111/ecc.13252 32495471

[30] Lan M, Zhang L, Zhang Y, Yan J. The relationship among illness perception, coping and functional exercise adherence in Chinese breast cancer survivors. J Adv Nurs. 2019;75(1):75–84. doi: 10.1111/jan.13832 30132970

[31] Karterud HN, Haavet OR, Risør MB. Social participation in young people with nonepileptic seizures (NES): A. Epilepsy & Behavior: E&B. 2016;57(Pt A):23–8. doi: 10.1016/j.yebeh.2016.01.009 26921594

[32] Berg P, Book K, Dinkel A, Henrich G, Marten-Mittag B, Mertens D, et al. Fear of progression in chronic diseases. Psychotherapie, Psychosomatik, Medizinische Psychologie. 2011;61(1):32–7. doi: 10.1055/s-0030-1267927 21120791

[33] Sharpe L, Michalowski M, Richmond B, Menzies RE, Shaw J. Fear of progression in chronic illnesses other than cancer: a systematic review and meta-analysis of a transdiagnostic construct. Health Psychol Rev. 2023;17(2):301–20. doi: 10.1080/17437199.2022.2039744 35132937

[34] Xiong J, Qin J, Zheng G, Gao Y, Gong K. The relationship between symptom perception and fear of progression in patients with chronic heart failure: a multiple mediation analysis. Eur J Cardiovasc Nur. 2023;22(6):638–46. doi: 10.1093/eurjcn/zvad024 36748202

[35] Reb AM, Borneman T, Economou D, Cangin MA, Patel SK, Sharpe L. Fear of Cancer Progression: Findings From Case Studies and a Nurse-Led Intervention. Clin J Oncol Nurs. 2020;24(4):400–8. doi: 10.1188/20.CJON.400-408 32678373 PMC8366305

[36] Wittwer A, Sponholz K, Frietsch JJ, Linke P, Kropp P, Hochhaus A, et al. Psychosocial distress in young adults surviving hematological malignancies: a pilot study. J Cancer Res Clin. 2023;149(9):5655–63. doi: 10.1007/s00432-022-04527-8 36527483 PMC10356626

[37] Broadbent E, Petrie KJ, Main J, Weinman J. The brief illness perception questionnaire. J Psychosom Res. 2006;60(6):631–7. doi: 10.1016/j.jpsychores.2005.10.020 16731240

[38] Broadbent E, Petrie KJ, Main J, Weinman J. The brief illness perception questionnaire. J Psychosom Res. 2006;60(6):631–7. doi: 10.1016/j.jpsychores.2005.10.020 16731240

[39] Mai Q, Xu S, Hu J, Sun X, Chen G, Ma Z, et al. The association between socioeconomic status and health-related quality of life among young and middle-aged maintenance hemodialysis patients: multiple mediation modeling. Front Psychiatry. 2023;14:1234553. doi: 10.3389/fpsyt.2023.1234553 37795510 PMC10546310

[40] Mehnert A, Herschbach P, Berg P, Henrich G, Koch U. [Fear of progression in breast cancer patients—validation of the short form of the Fear of Progression Questionnaire (FoP-Q-SF)]. Z Psychosom Med Psyc. 2006;52(3):274–88. 10.13109/zptm.2006.52.3.274 17156600

[41] Li B, Liu D, Zhang Y, Xue P. Stigma and related factors among renal dialysis patients in China. Front Psychiatry. 2023;14:1175179. doi: 10.3389/fpsyt.2023.1175179 37583843 PMC10423816

[42] Jessor R, Jessor SL. Problem behavior and psychosocial development: A longitudinal study of youth. New York:Academic Press. 1977;7(6):948–9.

[43] Liu Q, Zhang L, Xiang X, Mao X, Lin Y, Li J, et al. The influence of social alienation on maintenance hemodialysis patients’ coping styles: chain mediating effects of family resilience and caregiver burden. Front Psychiatry. 2023;14:1105334. doi: 10.3389/fpsyt.2023.1105334 37457762 PMC10342202

[44] Hayes AF, Rockwood NJ. Regression-based statistical mediation and moderation analysis in clinical research: Observations, recommendations, and implementation. Behav Res Ther. 2017;98:39–57. doi: 10.1016/j.brat.2016.11.001 27865431

[45] Preacher KJ, Kelley K. Effect size measures for mediation models: quantitative strategies for communicating indirect effects. Psychol Methods. 2011;16(2):93–115. doi: 10.1037/a0022658 21500915

[46] Karterud HN, Haavet OR, Risør MB. Social participation in young people with nonepileptic seizures (NES): A qualitative study of managing legitimacy in everyday life. Epilepsy & Behavior: E&B. 2016;57(Pt A):23–8. doi: 10.1016/j.yebeh.2016.01.009 26921594

[47] Hu N, Wang A, Chang T. Social support mediates the relationship between illness perception and psychosocial adaptation among young and middle-aged kidney transplant recipients in China. Front Psychol. 2023;14:1062337. doi: 10.3389/fpsyg.2023.1062337 36910788 PMC9998938

[48] Hagger MS, Orbell S. The common sense model of illness self-regulation: a conceptual review and proposed extended model. Health Psychol Rev. 2022;16(3):347–77. doi: 10.1080/17437199.2021.1878050 33461402

[49] Machado V, Botelho J, Proença L, Mendes JJ. Self-reported illness perception and oral health-related quality of life predict adherence to initial periodontal treatment. J Clin Periodontol. 2020;47(10):1209–18. doi: 10.1111/jcpe.13337 32592600

[50] Sir Ö, Özakgül A. Evaluation of the Perception of Illness and Quality of Life in Patients with Acute Myocardial Infarction. Turk Kardiyoloji Dernegi Arsivi: Turk Kardiyoloji Derneginin Yayin Organidir. 2022;50(3):209–16. doi: 10.5543/tkda.2022.21048 35450845

[51] Ośmiałowska E, Staś J, Chabowski M, Jankowska-Polańska B. Illness Perception and Quality of Life in Patients with Breast Cancer. Cancers. 2022;14(5). doi: 10.3390/cancers14051214 35267522 PMC8909179

[52] Sováriová Soósová M, Suchanová R, Parová V, Ulbrichtová A, Kopčová O, Rimárová K. Association Between Illness Perception and Adherence to Treatment in Slovak Patients With Hypertension: A Cross-sectional Study. The Journal of Cardiovascular Nursing. 2023;38(5):433–42. doi: 10.1097/JCN.0000000000000913 35420560

[53] Hagger MS, Koch S, Chatzisarantis NLD, Orbell S. The common sense model of self-regulation: Meta-analysis and test of a process model. Psychol Bull. 2017;143(11):1117–54. doi: 10.1037/bul0000118 28805401

[54] Cui C, Wang L, Wang X. Profiles of social constraints and associated factors among breast cancer patients: a latent profile analysis. Bmc Psychiatry. 2022;22(1):750. doi: 10.1186/s12888-022-04407-y 36451108 PMC9714186

[55] Fardell JE, Thewes B, Turner J, Gilchrist J, Sharpe L, Smith AB, et al. Fear of cancer recurrence: a theoretical review and novel cognitive processing formulation. Journal of Cancer Survivorship: Research and Practice. 2016;10(4):663–73. doi: 10.1007/s11764-015-0512-5 26782171

[56] Moussaoui LS, Claxton N, Desrichard O. Fear appeals to promote better health behaviors: an investigation of potential mediators. Health Psychol Behav. 2021;9(1):600–18. doi: 10.1080/21642850.2021.1947290 34285825 PMC8266257

[57] Loughan AR, Lanoye A, Aslanzadeh FJ, Villanueva AAL, Boutte R, Husain M, et al. Fear of Cancer Recurrence and Death Anxiety: Unaddressed Concerns for Adult Neuro-oncology Patients. J Clin Psychol Med S. 2021;28(1):16–30. doi: 10.1007/s10880-019-09690-8 31848927 PMC7461618

[58] Kuang X, Long F, Chen H, Huang Y, He L, Chen L, et al. Correlation research between fear of disease progression and quality of life in patients with lung cancer. Ann Palliat Med. 2022;11(1):35–44. doi: 10.21037/apm-21-2821 35144396

[59] Tauber NM, O’Toole MS, Dinkel A, Galica J, Humphris G, Lebel S, et al. Effect of Psychological Intervention on Fear of Cancer Recurrence: A Systematic Review and Meta-Analysis. Journal of Clinical Oncology: Official Journal of the American Society of Clinical Oncology. 2019;37(31):2899–915. doi: 10.1200/JCO.19.00572 31532725 PMC6823887

[60] Thiele S, Goebel S, Kröger N, Pedersen A. Fear of disease progression and relevant correlates in acute leukemia patients prior to allogeneic hematopoietic stem cell transplantation. Psycho-Oncology. 2020;29(8):1248–54. doi: 10.1002/pon.5397 32323380

[61] Frontini R, Sousa H, Ribeiro Ó, Figueiredo D. "What do we fear the most?": Exploring fears and concerns of patients, family. Scand J Caring Sci. 2021;35(4):1216–25. doi: 10.1111/scs.12940 33615525

[62] Song J, Cho E, Cho I, Lee D, Kim J, Kim H, et al. Mediating Effect of Intolerance of Uncertainty and Cancer-Related Dysfunctional Beliefs About Sleep on Psychological Symptoms and Fear of Progression Among Cancer Patients. Psychiat Invest. 2023;20(10):912–20. doi: 10.30773/pi.2023.0127 37899214 PMC10620329

[63] Chen Y, Lin C, Lee B. Relationships of illness representation and quality of life in patients with end-stage renal disease receiving haemodialysis. J Clin Nurs. 2020;29(19–20):3812–21. doi: 10.1111/jocn.15412 32644237

[64] Herzog K, Schepper F, Pletschko T, Herrmann J, Budich M, Christiansen H, et al. Illness perceptions, fear of progression and health-related quality of life during acute treatment and follow-up care in paediatric cancer patients and their parents: a cross-sectional study. Bmc Psychol. 2023;11(1):44. doi: 10.1186/s40359-023-01078-6 36782336 PMC9926758

[65] Shim E, Lee JW, Min YH. Does depression decrease the moderating effect of self-efficacy in the relationship between illness perception and fear of progression in breast cancer? Psycho-Oncology. 2018;27(2):539–47. doi: 10.1002/pon.4532 28816370

[66] Chen R, Yang H, Zhang H, Chen J, Liu S, Wei L. Fear of progression among postoperative patients with newly diagnosed lung cancer: a cross-sectional survey in China. Bmc Psychol. 2023;11(1):168. doi: 10.1186/s40359-023-01211-5 37217966 PMC10201766

[67] Aydın Sayılan A, Demir Doğan M. Illness perception, perceived social support and quality of life in patients with diagnosis of cancer. Eur J Cancer Care. 2020;29(4):e13252. doi: 10.1111/ecc.13252 32495471

[68] Liu Y, Wei M, Guo L, Guo Y, Zhu Y, He Y. Association between illness perception and health behaviour among stroke patients: The mediation effect of coping style. J Adv Nurs. 2021;77(5):2307–18. doi: 10.1111/jan.14761 33481272

[69] Shim HY, Cho MK. Factors influencing the quality of life of haemodialysis patients according to symptom cluster. J Clin Nurs. 2018;27(9–10):2132–41. doi: 10.1111/jocn.13904 28557301

[70] McLean RM, Xie Z, Nelson V, Nosa V, Thein H, Po’E-Tofaeono A, et al. Experiences of New Zealand Haemodialysis Patients in Relation to Food and Nutrition Management: A Qualitative Study. Nutrients. 2021;13(7). doi: 10.3390/nu13072299 34371809 PMC8308339

[71] Liu S, Wang Z, Zhang S, Xiao J, You L, Zhang Y, et al. The association between dose of hemodialysis and patients mortality in a prospective cohort study. Sci Rep-Uk. 2022;12(1):13708. doi: 10.1038/s41598-022-17943-0 35962178 PMC9374660

[72] Wen J, Fang Y, Su Z, Cai J, Chen Z. Mental health and its influencing factors of maintenance hemodialysis patients: a semi-structured interview study. Bmc Psychol. 2023;11(1):84. doi: 10.1186/s40359-023-01109-2 36978141 PMC10054072

[73] Nsamenang SA, Hirsch JK. Positive psychological determinants of treatment adherence among primary care patients. Primary Health Care Research & Development. 2015;16(4):398–406. doi: 10.1017/S1463423614000292 25158934

[74] Sels L, Tran A, Greenaway KH, Verhofstadt L, Kalokerinos EK. The social functions of positive emotions. Curr Opin Behav Sci. 2021;39:41–5. doi: 10.1016/j.cobeha.2020.12.009

[75] Salazar Kämpf M, Adam L, Rohr MK, Exner C, Wieck C. A Meta-Analysis of the Relationship Between Emotion Regulation and Social Affect and Cognition. Clin Psychol Sci. 2023;11(6):1159–89. doi: 10.1177/21677026221149953

[76] English T, John OP, Srivastava S, Gross JJ. Emotion Regulation and Peer-Rated Social Functioning: A Four-Year Longitudinal Study. J Res Pers. 2012;46(6):780–4. doi: 10.1016/j.jrp.2012.09.006 23471162 PMC3587109

[77] Komase Y, Watanabe K, Hori D, Nozawa K, Hidaka Y, Iida M, et al. Effects of gratitude intervention on mental health and well-being among workers: A systematic review.; 2021. p. e12290. 10.1002/1348-9585.12290 PMC8582291 34762326

[78] Medvedev ON, Landhuis CE. Exploring constructs of well-being, happiness and quality of life. Peerj. 2018;6:e4903. doi: 10.7717/peerj.4903 29876148 PMC5985772

[79] Sadeghi Akbari A, Cheraghi MA, Kazemnejad A, Nomali M, Zakerimoghadam M. Effect of Illness Perception Correction—Based Educational Program on Quality Of Life and Self- Care in Patients with Heart Failure: a Randomized Controlled Trial. Journal of Caring Sciences. 2019;8(2):89–93. doi: 10.15171/jcs.2019.013 31249818 PMC6589485

[80] Minjie Z, Zhijuan X, Xinxin S, Xinzhu B, Shan Q. The effects of cognitive behavioral therapy on health-related quality of life, anxiety, depression, illness perception, and in atrial fibrillation patients: a six-month longitudinal study. Bmc Psychol. 2023;11:431. doi: 10.1186/s40359-023-01457-z 38062475 PMC10704769

[81] Clark DA. Cognitive Restructuring.; 2013. p. 1–22.

[82] Barello S, Anderson G, Acampora M, Bosio C, Guida E, Irace V, et al. The effect of psychosocial interventions on depression, anxiety, and quality of life in hemodialysis patients: a systematic review and a meta-analysis. Int Urol Nephrol. 2023;55(4):897–912. doi: 10.1007/s11255-022-03374-3 36180655 PMC10030538

[83] Yang H, Qi L, Pei D. Effect of psychosocial interventions for depression in adults with chronic kidney disease: a systematic review and meta-analysis. Bmc Nephrol. 2024;25(1):17. doi: 10.1186/s12882-023-03447-0 38200465 PMC10782786

[84] Gentry MT, Lapid MI, Clark MM, Rummans TA. Evidence for telehealth group-based treatment: A systematic review. J Telemed Telecare. 2019;25(6):327–42. doi: 10.1177/1357633X18775855 29788807

[85] Haleem A, Javaid M, Singh RP, Suman R. Telemedicine for healthcare: Capabilities, features, barriers, and applications. Sensors International. 2021;2:100117. doi: 10.1016/j.sintl.2021.100117 34806053 PMC8590973

[86] Eysenbach G, Powell J, Englesakis M, Rizo C, Stern A. Health related virtual communities and electronic support groups: systematic review of the effects of online peer to peer interactions. Bmj (Clinical Research Ed.). 2004;328(7449):1166. doi: 10.1136/bmj.328.7449.1166 15142921 PMC411092

[87] Taylor F, Taylor C, Baharani J, Nicholas J, Combes G. Integrating emotional and psychological support into the end-stage renal disease pathway: a protocol for mixed methods research to identify patients’ lower-level support needs and how these can most effectively be addressed. Bmc Nephrol. 2016;17(1):111. doi: 10.1186/s12882-016-0327-2 27484760 PMC4971672

[88] Goh ZS, Griva K. Anxiety and depression in patients with end-stage renal disease: impact and management challenges—a narrative review. Int J Nephrol Renov. 2018;11:93–102. doi: 10.2147/IJNRD.S126615 29559806 PMC5856029

